# Endoscopic Ultrasound-Guided Transesophageal Drainage of a Posterior Mediastinal Abscess Secondary to Vertebral Osteomyelitis

**DOI:** 10.14309/crj.0000000000002218

**Published:** 2026-06-22

**Authors:** Joelle Sleiman, Ali Sohail, Hassan Al Moussawi, Youssef El Douaihy

**Affiliations:** 1 Department of Medicine, Northwell Staten Island University Hospital, Staten Island, NY; 2 Division of Gastroenterology, Northwell Staten Island University Hospital, Staten Island, NY

**Keywords:** endoscopic ultrasound, mediastinal abscess, vertebral osteomyelitis, transesophageal drainage, interventional endoscopy

## Abstract

Mediastinal abscesses are life-threatening infections traditionally managed with surgical or percutaneous drainage. Endoscopic ultrasound (EUS)-guided drainage has emerged as a minimally invasive alternative for deep-seated collections, but experience with mediastinal abscesses remains limited. We present a case of successful EUS-guided transesophageal drainage of a posterior mediastinal abscess complicating vertebral osteomyelitis. A 50-year-old man with T3-T4 vertebral osteomyelitis and a posterior mediastinal abscess underwent EUS-guided drainage after percutaneous access was deemed unfeasible. A 19-gauge needle was used for aspiration, followed by guidewire placement and double-pigtail stent insertion. Technical success was achieved with placement of a 7-French × 4-cm double-pigtail stent. Blood and fluid cultures grew methicillin-sensitive *Staphylococcus aureus*. Complete radiographic resolution was confirmed at 3 days, the stent was removed, and an esophagram on the following day demonstrated no leak. The patient was discharged on a 6-week course of intravenous antibiotics with resolution of all clinical symptoms. EUS-guided drainage with short-interval double-pigtail stent placement is a safe and effective minimally invasive approach for posterior mediastinal abscesses when percutaneous access is not feasible.

## INTRODUCTION

Mediastinal abscesses are life-threatening infections with mortality rates ranging from 1.8% to 35%, and outcomes are heavily influenced by the cause and timing of treatment. These collections typically arise from esophageal perforation, descending necrotizing mediastinitis, postsurgical adverse events, or hematogenous seeding from vertebral osteomyelitis.^1^ Traditional management has relied on surgical drainage by thoracotomy or video-assisted thoracoscopic surgery, complemented by percutaneous computed tomography (CT)-guided drainage when anatomically feasible.^2^ However, anatomic constraints may preclude this approach in mediastinal collections.

The emergence of therapeutic endoscopic ultrasound (EUS) has transformed the management of deep-seated fluid collections. EUS offers distinct advantages, including real-time visualization of vascular structures, placement of internal drainage catheters to avoid external catheters, and access to anatomically challenging locations. While EUS-guided drainage has been established for intra-abdominal and pelvic collections, the literature on mediastinal collections, especially posterior mediastinal collections, is limited due to the challenging anatomy and proximity to critical neurovascular structures.^3,4^

We present successful EUS-guided drainage of a paravertebral mediastinal abscess using double-pigtail stent placement, demonstrating the technical feasibility and clinical efficacy of this minimally invasive approach.

## CASE REPORT

A 50-year-old man presented to the emergency department with severe mid-upper back pain. Thoracic spine CT demonstrated discitis and osteomyelitis affecting T3 and T4 vertebrae, with cortical destruction and lytic changes. A rim-enhancing collection in the posterior mediastinal space, anterior to the T3-T4 vertebral bodies, was observed, causing anterior displacement of the trachea and esophagus. Interventional radiology consultation determined that percutaneous drainage was not feasible due to a limited window. Empiric intravenous antibiotic therapy was initiated on the day of admission, and the patient was referred for EUS evaluation for abscess drainage given the size and purulent character of the collection together with an unresolved infectious source. Drainage was performed approximately 48 hours after antibiotic initiation.

**Figure 1.**
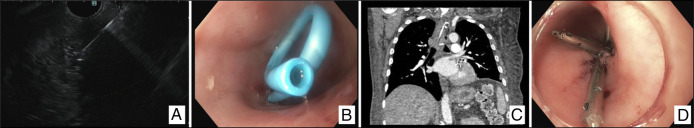
Endoscopic ultrasound-guided transesophageal drainage of a posterior mediastinal abscess. (A) Endoscopic ultrasound image with the double-pigtail stent (arrow) traversing the esophageal wall into the abscess cavity (asterisk). (B) Endoscopic view of the proximal end of the double-pigtail stent (arrow) within the esophageal lumen. (C) Axial computed tomography image of the chest demonstrating the double-pigtail stent (arrow) extending from the esophagus into the soft tissues of the posterior mediastinum, with marked reduction of the previously seen abscess cavity. (D) Endoscopic view of the esophageal entry site closed with through-the-scope clips (arrows) following stent removal.

A linear echoendoscope was advanced to the proximal paraesophageal region. At 25 cm from the incisors, a 30 × 31 mm heterogeneous collection was identified, consistent with the perivertebral abscess described on CT imaging. Fine-needle aspiration was performed using a 19-gauge needle, with color Doppler interrogation to avoid intervening vasculature. Five milliliters of purulent material were aspirated and sent for microbiological analysis. A guidewire was advanced into the collection under EUS guidance. A cystotome was then advanced over the guidewire, with proper positioning confirmed by EUS. The linear echoendoscope was then exchanged for a standard forward-viewing upper endoscope over the indwelling guidewire to permit direct en-face visualization for precise stent deployment, given the oblique optical axis and narrower working channel of the linear echoendoscope. A 7-French × 4-cm double-pigtail plastic stent was deployed with the distal end within the abscess cavity and the proximal end in the esophageal lumen. Stent position was confirmed by endoscopy, EUS, and chest CT (Figure 1).

Blood and fluid cultures subsequently grew methicillin-sensitive *Staphylococcus aureus*, and antibiotic therapy was directed with intravenous nafcillin, which was later transitioned to cefazolin. Transesophageal echocardiography demonstrated no valvular vegetations; the vertebral osteomyelitis with contiguous paravertebral extension was therefore considered the most likely source of bacteremia.

The patient was maintained on strict nil per os (NPO) status with the stent in place, with no enteral or parenteral nutritional support required given the brief drainage interval. A repeat CT scan on postprocedure day 3 demonstrated complete resolution of the collection, prompting an early follow-up esophagogastroduodenoscopy during which the double-pigtail stent was removed using rat-tooth forceps. The esophageal entry site was closed using through-the-scope clips (Figure 1). On the following day, a fluoroscopic esophagram demonstrated no contrast extravasation; the patient was advanced from ice chips to a clear liquid diet and then to a regular oral diet, all of which were tolerated without difficulty. The patient tolerated the intervention well, with resolution of all clinical symptoms and signs of infection, and was discharged on a 6-week course of intravenous antibiotics.

## DISCUSSION

This case demonstrates successful EUS-guided drainage of a posterior mediastinal abscess complicating vertebral osteomyelitis, adding to the limited but growing evidence supporting this minimally invasive approach. While systematic data exist on EUS-guided drainage of pelvic and pancreatic collections, published experience with mediastinal abscesses remains limited, largely confined to case reports and small series.^5–7^

Mediastinal abscesses occur through various mechanisms, with postcardiac surgery mediastinitis representing the most common etiology (0.25%–5% of cardiac surgeries), followed by descending necrotizing mediastinitis and esophageal perforation.^8,9^ Vertebral osteomyelitis with paravertebral extension, as observed in our patient, represents a less common but important cause, with *Staphylococcus aureus* identified as the predominant pathogen in approximately half of cases.^4^ Thoracic vertebral involvement may allow infection to extend anteriorly into the posterior mediastinum, creating collections that are anatomically difficult to access using conventional surgical or percutaneous approaches. The annual incidence of vertebral osteomyelitis approximates 2.4 cases per 100,000 persons, with mediastinal extension occurring in a subset of thoracic vertebral involvement.^10^ In our patient, blood-culture-confirmed methicillin-sensitive *Staphylococcus aureus* bacteremia in the absence of valvular vegetations on transesophageal echocardiography supported a presumed hematogenous origin from the vertebral focus, with contiguous extension producing the paravertebral mediastinal collection.

The decision to pursue interventional drainage rather than antibiotic therapy alone deserves explicit consideration. Antimicrobial monotherapy may suffice for small, well-contained paravertebral collections in clinically improving patients without neurologic compromise. Current guidelines on native vertebral osteomyelitis, however, recommend image-guided or surgical source control for larger collections, those producing neurologic deficit, or those failing to respond to appropriate antimicrobial therapy.^11^ In our patient, the dimensions of the collection (30 × 31 mm), the frankly purulent character of the aspirate, and the active spondylodiscitis as an ongoing infectious source supported a combined antimicrobial and drainage strategy.

When percutaneous drainage is not feasible due to limited access windows or proximity to critical structures, EUS-guided drainage offers a minimally invasive alternative. Limited literature exists on EUS-guided drainage of mediastinal collections; most studies support percutaneous drainage with a 95.6% clinical success rate, though with a median drainage duration of 13.6 days.^2^ For comparison, EUS-guided drainage of upper abdominal abscesses demonstrates comparable success rates (88.9%) with similar safety profiles.^12^ For pelvic collections, systematic reviews demonstrate EUS-guided drainage achieving 92% clinical success rates (95% confidence interval: 87%–98%) with 100% technical success in 135 patients.^3^ Current evidence from pancreatic fluid collections strongly supports transgastric routes.^13^ Although these data primarily derive from intra-abdominal collections, the same technical principles may be applied to mediastinal abscesses when a safe endosonographic window is present. The use of 19-gauge needles provides superior guidewire compatibility, while real-time Doppler guidance allows for careful avoidance of intervening vascular structures. These features make EUS particularly advantageous in the mediastinum, where major vascular structures are frequently encountered.

Within the spectrum of EUS-guided interventions, single-session needle aspiration was considered but felt to be inadequate for sustained source control in our patient. Although the collection measured only 30 × 31 mm, the thick purulent character of the aspirate, combined with the unresolved primary focus in the adjacent vertebral bodies, conferred a substantial risk of reaccumulation following one-time aspiration alone. Internal transluminal stenting permitted continuous decompression of the cavity, a defined endpoint for source control through serial imaging, and allowed safe stent removal once radiographic resolution was confirmed. This consideration is consistent with strategies employed for other deep-seated abscesses with persistent inflammatory drivers and with case reports of mediastinal collections in which simple aspiration has been associated with recurrence.^5–7^

Stent selection and duration represent additional important considerations. Selection between plastic double-pigtail stents and lumen-apposing metal stents depends on collection characteristics; recent studies suggest plastic stents remain appropriate for smaller collections with lower tissue ingrowth risk, as in our case.^14^ Optimal stent duration for mediastinal collections remains undefined, though evidence from pancreatic collections suggests no difference in adverse event rates between early (within 3–4 weeks) and late (>4 weeks) lumen-apposing metal stents removal, supporting individualized timing based on clinical and radiographic response.^15^ In the present case, complete radiographic resolution at 3 days justified early stent removal, minimizing the duration of an indwelling foreign body across the esophageal wall and limiting the period of strict NPO management. The exchange from the linear echoendoscope to a forward-viewing upper endoscope for stent deployment is a commonly employed technique that combines the precise needle and guidewire control of EUS with the en-face visualization and larger working channel required for confident stent positioning.

The integration of advanced closure techniques represents an important technical consideration. While some series report successful outcomes without formal closure of the drainage site, we employed clip closure to minimize fistula risk. Recent advances in endoscopic closure, including over-the-scope clips and suturing systems, provide robust options for managing the esophageal entry site following stent removal.^16,17^ In our patient, postremoval fluoroscopic esophagram confirmed integrity of the closure before oral diet advancement, an additional precaution that may further reduce the risk of clinically significant leak in this anatomically high-stakes location.

Appropriate patient selection remains critical for successful outcomes with EUS-guided drainage. This approach appears most suitable for mediastinal collections with an adequate acoustic window and absence of intervening vascular structures. The proximity to the esophagus in our case provided ideal conditions for transesophageal drainage. Contraindications include coagulopathy, hemodynamic instability requiring emergent surgical intervention, and collections not accessible via transgastric or transesophageal routes.

This case supports the use of EUS-guided drainage as a safe and efficacious approach for posterior mediastinal abscesses. The successful management of a vertebral osteomyelitis-related collection expands the reported applications of this technique. The complete clinical and radiographic resolution achieved with an abbreviated 3-day drainage period and minimal adverse events suggests that endoscopic drainage may represent a valuable alternative to more invasive surgical approaches in appropriately selected cases. Prospective studies are warranted to further define patient selection criteria, optimal stent type and duration, and long-term outcomes.

## DISCLOSURES

Author contributions: J. Sleiman: drafting of the article, critical revision of the article for important intellectual content, final approval of the article. A. Sohail: drafting of the article, critical revision of the article for important intellectual content, final approval of the article. HA Moussawi: conception and design, analysis and interpretation of the data, critical revision of the article for important intellectual content, final approval of the article. YE Douaihy: conception and design, analysis and interpretation of the data, critical revision of the article for important intellectual content, final approval of the article. A. Sohail is the article guarantor.

Financial disclosure: None to report.

Previous presentation: This case was presented as an abstract at the American College of Gastroenterology Annual Scientific Meeting, October 25–30, 2024; Philadelphia, Pennsylvania.

Informed consent was obtained for this case report.

## ABBREVIATIONS:

CT: computerized tomography
EUS: endoscopic ultrasound
LAMS: lumen-apposing metal stent
NPO: nil per os
VATS: video assisted thoracoscopic surgery
